# How Accurate Can UWB and Dead Reckoning Positioning Systems Be? Comparison to SLAM Using the RPLidar System

**DOI:** 10.3390/s20133761

**Published:** 2020-07-05

**Authors:** Damian Grzechca, Adam Ziębiński, Krzysztof Paszek, Krzysztof Hanzel, Adam Giel, Marcin Czerny, Andreas Becker

**Affiliations:** 1 Department of Electronics, Electrical Engineering and Microelectronics, Silesian University of Technology, Akademicka 16, 44-100 Gliwice, Poland; Krzysztof.Paszek@polsl.pl (K.P.); Krzysztof.Hanzel@polsl.pl (K.H.); gielupl@gmail.com (A.G.); czernymarcin@gmail.com (M.C.); 2 Department of Distributed Systems and Informatic Devices, Silesian University of Technology, Akademicka 16, 44-100 Gliwice, Poland; Adam.Ziebinski@polsl.pl; 3 Faculty of Information Technology, University of Applied Science and Arts, Sonnenstr. 96, 44139 Dortmund, Germany; Andreas.Becker@fh-dortmund.de

**Keywords:** dead reckoning, UWB, positioning system, RPLidar, SLAM

## Abstract

This paper compares two positioning systems, namely ultra-wideband (UWB) based micro-location technology and dead reckoning and a RPLidar based simultaneous localization and mapping (SLAM) solution. This new approach can be used to improve the quality of the positioning system and increase the functionality of advanced driver assistance systems (ADAS). This is achieved by using stationary nodes and UWB tags on the vehicles. Thus, the redundancy of localization can be achieved by this approach, e.g., as a backup to onboard sensors like RPlidar or radar. Additionally, UWB based micro-location allows additional data channels to be used for communication purposes. Furthermore, it is shown that the regular use of correction data increases UWB and dead reckoning accuracy. These correction data can be based on onboard sensors. This shows that it is promising to develop a system that fuses onboard sensors and micro-localization for safety-critical tasks like the platooning of commercial vehicles.

## 1. Introduction

The current massive car traffic requires changes in methods of transport utilization. One of the ways to increase the efficiency of transport utilization is the usage of an automatic highway system (AHS) [1], including automated platooning [2]. To be able to use this technology, it is necessary to be able to determine the distance to the preceding vehicle [3] and to predict the path it moves on. Modern cars use, e.g., lidar technology to ensure this purpose. Thanks to the reflection of the laser beam, it is able to provide the distance to the object. However, this solution has its drawbacks, not only related to the reflection itself (associated, for example, with the surface or the need to mount at a certain height), but also with the possibility of distortions, especially in the form of weather conditions.

However, there should be a way to ensure maximum security and, at the same time, take advantage of such things as increasing traffic safety on roads and allow to reduce fuel consumption [4]. Positioning methods should also, in the future, enable the implementation of autonomous driving [5], which is associated with many challenges not only from the point of view of algorithmics, but also the accuracy of acquired position data. Often, in such systems, various types of measurements on the basis of radar and laser sensors and advanced solutions like ADAS modules [6] which allow to monitor the environment are used. Many positioning systems should be considered in the positioning process. All their advantages (e.g., high accuracy, high data rate) should be used to eliminate all disadvantages (low accuracy, low data rate, costs, precision) of an individual system [7]. Some of them used UWB [8] and inertial measurement unit (IMU), e.g., for range-based cooperative localization method [9].

The movement of vehicles close to each other with high speed influences the appearance of additional dangerous factors. Therefore, the use of a data fusion algorithm [10,11] that will take into account the measurement of speed [12] and distance to the preceding car and other significant factors such as recognized road signs [13,14], road infrastructure [15], and driver [16] or system behavior is essential [17]. Additionally, the time to retrieve information from the environment by sensors and fast processing of those data is important. As the speed increases, it becomes crucial to receive information as soon as possible. The current verification of the measurement and control modules to ensure their correct operation is also important.

Information about, e.g., traffic, regulations, and speed limits, can be obtained from the UWB infrastructure, which allows to send additional data in a datagram. Communication between vehicles [18,19] is also possible, but it has to be secure. In particular, information about the vehicle’s current and expected maneuvers and the speed of the vehicle can be useful to avoid collisions. Communication with infrastructure and other road users, e.g., cyclists and pedestrians [20], can reduce the number of violent and dangerous maneuvers and situations. So, the integration of several technologies [21] is necessary for safe movement [22,23] on public roads.

The main goal of this research is to obtain the most accurate position by the use of data fusion using the UWB system, IMU, and RPLidar. In this article, low-cost solutions based on an encoder, i.e., three-dimensional attitude heading reference system (myAHRS+)(dead reckoning), 360-degree 2D laser scanner (RPLidar), and positioning based on UWB system (Pozyx), are presented. The following sections first introduce the architecture of the remote-controlled platform, parameters, as well as pros and cons of each used positioning systems. Next, the trilateration method and research environment are described. Finally, the conducted research with results and discussion are presented. Such a solution could be used to improve the quality of the positioning system and increase the functionality of ADAS modules.

## 2. The Architecture of the Remote-Controlled Platform

**Figure 1:**
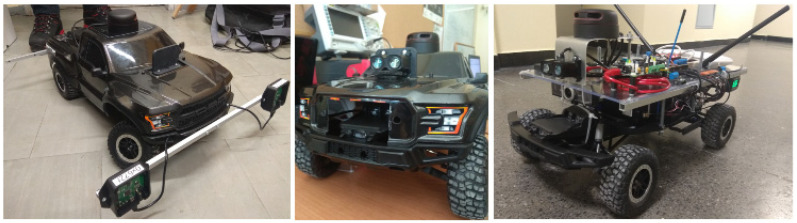
The evolution of the mobile platform used in tests.

One of the ways to test positioning systems in real-world scenarios is to use them on mobile platforms, that allow collecting data from sensors and steering indoors. Because of that, a popular radio-controlled (RC) platform model was equipped with, among others, single-board computers, microcontrollers, sensors, communication devices, and a power supply. The final platform for the research purpose is presented in Figure 1.

**Figure 2:**
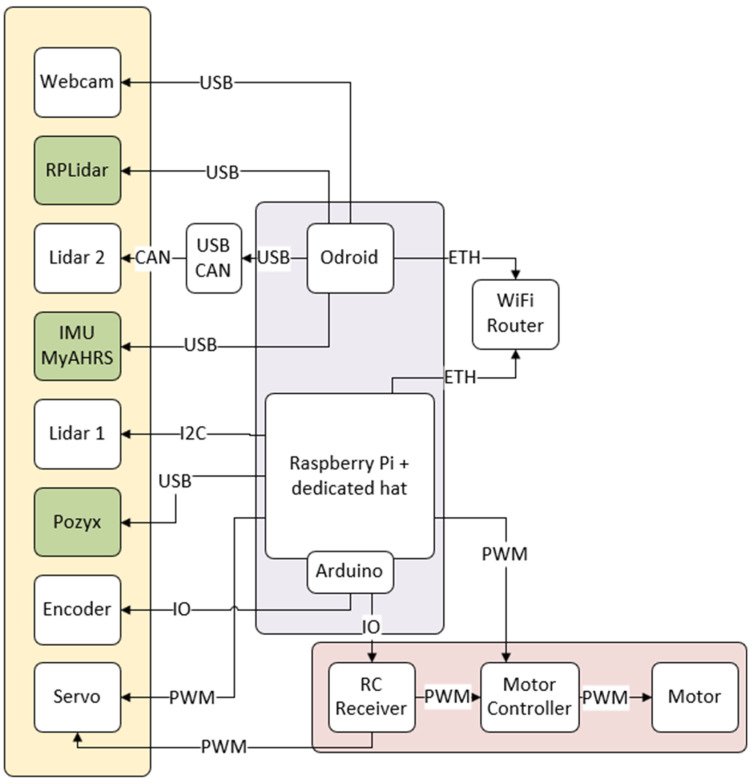
The diagram of connections between used components.

The diagram of connections between used components is presented in Figure 2. Due to hardware limitations and data transfer components like camera, RPLidar and IMU have been separated from the UWB modules (POZYX on the schematic diagram) and encoders. A Raspberry Pi computer was used to remote control of the vehicle, control the motor and steering servo by pulse width modulation (PWM signal). Additionally, it collects sensors data, particularly from an encoder (possible because of intermediary Arduino) and Pozyx (based on the Dwcawave DW1000) [24]. The Odroid computer was used to collect data from other sensors, like Lidars (Garmin LiteV3 HP, Lidar Continental, RPlidar), video camera, and IMU myAHRS+. These two computers were connected to each other by Ethernet cables to a Wi-Fi router that could also be used to make a wireless connection with a personal computer to monitor the system work.

**Figure 3:**
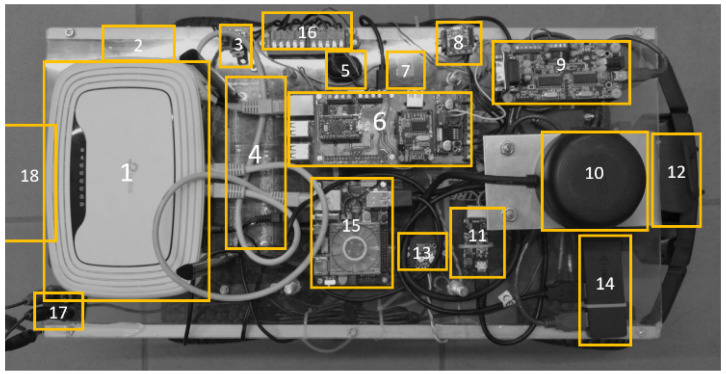
The arrangement of individual modules on the surface of the mobile platform.

One can notice that the platform has devices on two levels. The upper level shown in Figure 3 are as follows: (1) Wi-Fi router, for connecting with operator computer, (2) voltmeter, for battery condition observation, (3) 9 V voltage regulator, for supplying Wi-Fi router, (4) battery pack, for supplying the steering servo and motor on the platform, (5) power switch, (6) Raspberry Pi with a dedicated hat, for collecting data from sensors and controlling the platform, (7) 5 A fuse, (10) RPLidar, rotating Lidar dedicated to scanning the area, (11) USB/UART converter, for communication between Odroid XU 4 and Raspberry Pi, (13) stepdown voltage converter, for supplying sensors and computers on the platform, (14) USB hub—expanding the available USB ports, (15) Odroid XU4, for collecting data from RPLidar and controlling the platform, (16) connector, (17) external power supply, an alternative to the battery pack when the platform is not in motion, and (18) a UWB anchor, for positioning of the platform (Pozyx system).

**Figure 4:**
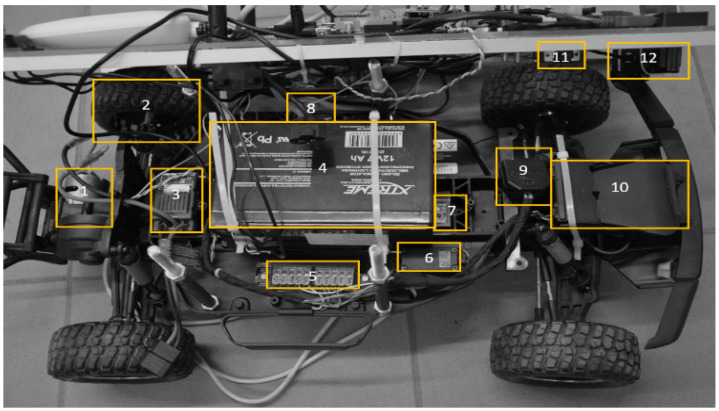
The arrangement of individual modules below the top board of the mobile platform.

The lower-level components are shown in Figure 4: (1) motor, (2) encoder, to measure the speed of the platform, (3) motor controller, (4) 12 V battery, for powering the motor, (5) connector, (6) servo, (7) IMU myAHRS+, for collecting data about acceleration and orientation, (8) RC receiver, for manual remote control, and (12) a USB webcam.

The chosen parameters of positioning subsystems or devices used in the RC platform and related systems are listed in the Table 1 Advantages, disadvantages and parameters related to devices used in the RC platform and related systems.

## 3. The UWB Positioning System

UWB is one of the most promising technologies for position determination and allows object tracking. This technology is still very actively researched and developed. UWB is a radio communication technique involving the transmission of very short radio pulses, which allows for the transmission of large amounts of data (up to 1.6 Gb/s) over a short distance (up to 10 m).

The basic feature that distinguishes this technology from conventional radio communication is the fact that conventional systems use signal amplitude, phase, or frequency modulation. The UWB sends information generating impulses of constant amplitude, with a time regime. Further, it uses a wideband for implementing time modulation. The system offers distance measurements based on the time of flight (ToF) method. It enables compensation of the multipath propagation and allows the use of UWB as positioning technology.

**Figure 5:**
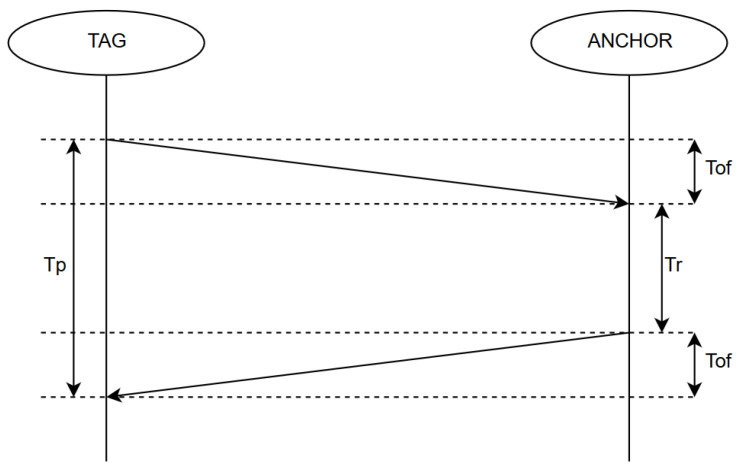
The principle of determining the relative position between anchor and tag.

The distance between the transmitter and receiver (tag and anchor) could be determined by measuring the time. The principle of determining the relative position between anchor and tag is presented in Figure 5. Given the exact time of sending the frame, the exact time of receiving the frame, and the speed of the signal (speed of light), the distance between the transmitter and receiver can be determined. Then, the trilateration algorithm [25,26] is used. Three reference points allow to calculate the position of the object in a two-dimensional plane. The transmitter sends a frame containing the moment of sending, while the receiver receives the frame and sends it back to the tag, which calculates the distance between the receiver and the transmitter using the ToF and speed of the wave propagation (speed of light).

**Figure 6:**
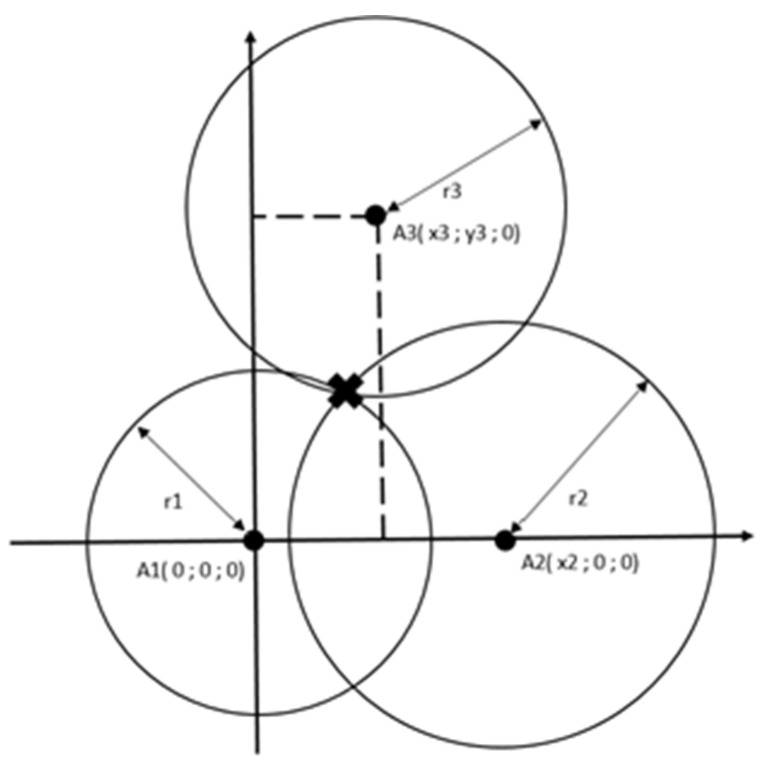
An example of trilateration.

According to Fang’s method [27], one sphere center is placed at the origin of the elevated plane, the second along with the x-axis, and the third orthogonally to the previous two, as presented in Figure 6.

Equations below depict anchor coordinates. Starting from points A1, A2, and A3 given by (1),

(1) $$A1\left(0;0;0\right)A2\left(x_{2};0;0\right)A3\left(x_{3};y_{3};0\right)$$

and the radius of the circles (2)

(2) $$\begin{array}{c} r_{1}^{2}=x^{2}+y^{2}+z^{2},\\ r_{2}^{2}={\left(x-x_{2}\right)}^{2}+y^{2}+z^{2},\\ r_{3}^{2}={\left(x-x_{3}\right)}^{2}+{\left(y-y_{3}\right)}^{2}+z^{2}, \end{array}$$

*x*, *y*, and, *z* can be determined:

(3) $$\begin{array}{c} x_{p}=\frac{r_{1}^{2}-r_{2}^{2}+x_{2}^{2}}{2x_{2}}\\ y_{p}=\frac{r_{1}^{2}-r_{3}^{2}+x_{3}^{2}+y_{3}^{2}-\left(2x_{3}x_{p}\right)}{2y_{3}}\\ z=\sqrt{r_{1}^{2}-x_{p}^{2}-y_{p}^{2}} \end{array}$$

## 4. Dead Reckoning

Dead reckoning is based on another approach [28].The current position of the object ($x_{t},\;y_{t}$) is determined based on the known position in the past $\left(x_{t-1},\;y_{t-1}\right)$, the direction (given by yaw angle α) and speed (v) with which it moves [29]. IMUs with encoders (odometry) usually acquire it. This technique requires knowledge of the initial position (x(0), y(0)), which should be as accurate as possible. The speed of the platform is calculated based on encoder readings (4). The wheel rotation was measured with an accuracy of 10 deg. Then, in the subsequent moments of time, the travelled distance (s) (5) and direction of the movement (α) are used for new position calculation (6). Each subsequent update position is burdened with an error that is accumulated (by the data source to the calculated speed and direction of movement of the subject).

(4) v(t)=2πr·θ·t 

where r refers to platform wheels radius, θ, the wheel rotation angle, and t the time elapsed since the previous measurements reading.

(5) $$s=\int_{t-1}^{t}v\left(t\right)dt$$

(6) $$\{\begin{array}{c} x_{t}=x_{t-1}+s\cos\left(\alpha \right)\\ y_{t}=y_{t-1}+s\sin\left(\alpha \right) \end{array}\;$$

**Figure 7:**
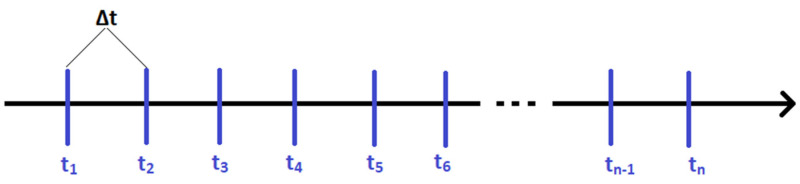
The sample route of the vehicle along with corrections of position from the UWB system or dead reckoning based on the readings (corrections) from the Lidar every Δt = {1 s or 3 s}.

An additional positioning method should be used to improve the accuracy and reduce the accumulated error of the position obtained by dead reckoning. This can be achieved by systematically updating the current position (initial position for dead reckoning) by the system with higher accuracy (see Figure 7). The data rate of that positioning system can be lower than that of the IMU/myAHRS+.

A new accurate position obtained from Lidar (or another high accurate positioning system) can be used as a new initial point for the dead reckoning (or another system with high data rate but lower accuracy), so the accumulated error is removed.

## 5. The Research Environment

The second more accurate system is needed to evaluate the system under test. RPLidar and information from encoders are used as a reference system (system with greater accuracy) to validate the UWB positioning system and dead reckoning. The main advantages of RPLidar are high accuracy, resolution, and relatively fast acquisition time. The stationary and characteristic reference points can be used to determine the accurate position of the mobile platform in a local coordinate system. These points can be detected by scanning the area surrounding the mobile platform by making use of a rotating lidar scanner (RPLidar). If these points are fixed, then by measuring differences in the historic relative position of these points to the moving mobile platform, the change of the vehicle orientation and position can be determined.

The UWB positioning system is the main core of this research. The applied UWB system consists of a set of anchors (reference points) and tags. The purpose of this system is to determine the position of the tags with respect to known anchors positions. All components are based on the UWB system, incorporating Decawave DWM1000 and STM microcontrollers. Functions the anchor or tag can be assigned to devices as desired. Differences between components do not affect their basic functionality determining position and distance.

A robotic operating system (ROS) was used for logging and processing data. It is a simple way to create robotics applications with many off-the-shelf nodes with good documentation. ROS applications allow logging data from multiple sources and timestamp them. The Hector SLAM package was used to create a reference path based on RPLidar.

**Figure 8:**
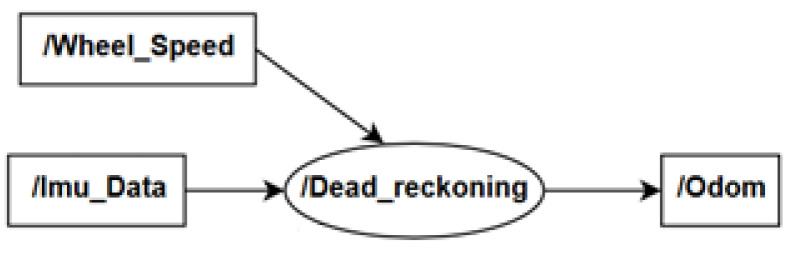
The ROS odometry topics structure.

The graph ROS odometry topic structure is presented in Figure 8. The Node/Dead_reckoning subscribes/Wheel_Speed topic with wheel speed data and /Imu_Data topic with yaw rate data. The output was published by Odom topic and contains position x and y in meters.

**Figure 9:**
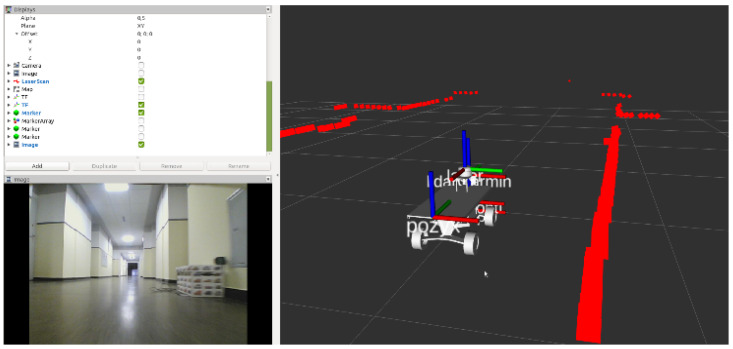
The visualization of the lidar scan points cloud and the generated map of the examined corridor using RviZ tool.

The ROS RviZ tool was used to visualize the state of the mobile platform in a three-dimensional environment. It allows displaying 3D models, point clouds, markers, creating maps, paths, trajectories, etc. All this is based on the mechanisms of publishing and subscribing to relevant types of messages. It gives a spectacular visual effect and allows for the initial verification of the measurements and their reference to reality. Figure 9 presents a visualization of the lidar scan points cloud and the generated map of the examined corridor. In the bottom left corner, the view from a camera connected to the mobile platform is presented, so it is easier to recognize the observed environment.

Lidar was used to create the map and determine the position, i.e., the SLAM technique. For the tests, Hector SLAM, one of the most popular SLAM implementations, was used. Hector SLAM was created at the University of Darmstadt [30]. The output is a map and the position of the object. The positions determined using this method will be the references for the other positioning methods. Mapping and location errors are cumulative. Incorrect estimation of the position translates into incorrect positioning of objects on the map. However, incorrect determination of the position of objects makes it difficult to determine its own position changes. The accumulation of errors can contribute to incorrect creation of a map in the mobile platform’s memory, not related to reality.

**Figure 10:**
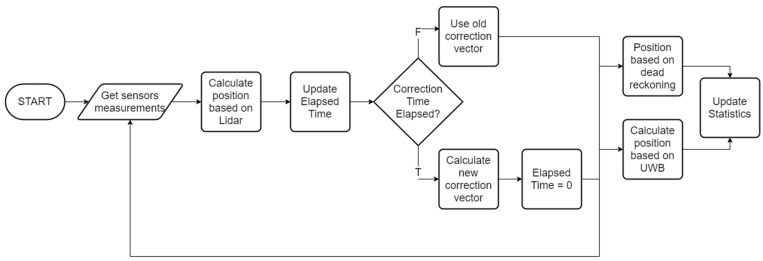
Block diagram of data flow in the prepared algorithm.

The positioning algorithms (presented in Figure 10) is based on taking measurements from Lidar, IMU, encoder and UWB system. In a given time interval the correction vector is calculated based on the current reference position from lidar and position indicated by UWB/dead reckoning values. The localization is determined with current measurements from UWB/dead reckoning and the last calculated correction vector.

**Figure 11:**
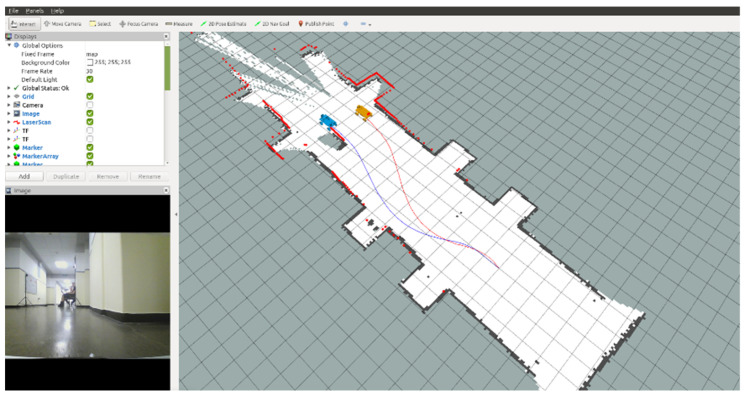
**The** comparison of SLAM (blue path) and dead reckoning (red path).

Data from Lidar were used as a reference for other positioning methods. All information about position obtained from the UWB system or dead reckoning was compared to the reference path determined by Lidar and Hector SLAM. The example of a comparison of two approaches (SLAM and dead reckoning) is presented in Figure 11. All the sensors were mounted on a built RC model. The distances between the sensors’ mounting points are taken into account when calculating the position of the object. Data from tests were logged using a rosbag file, which contains data with timestamps.

The goal of the research was to compare two methods of object positioning:

- Dead reckoning based on the data from myAHRS+ (angular acceleration) and from the encoder mounted on the rear wheel (speed of the vehicle),
- UWB (position in local reference system) with SLAM based on RPLidar data (for environment scanning).

**Figure 12:**
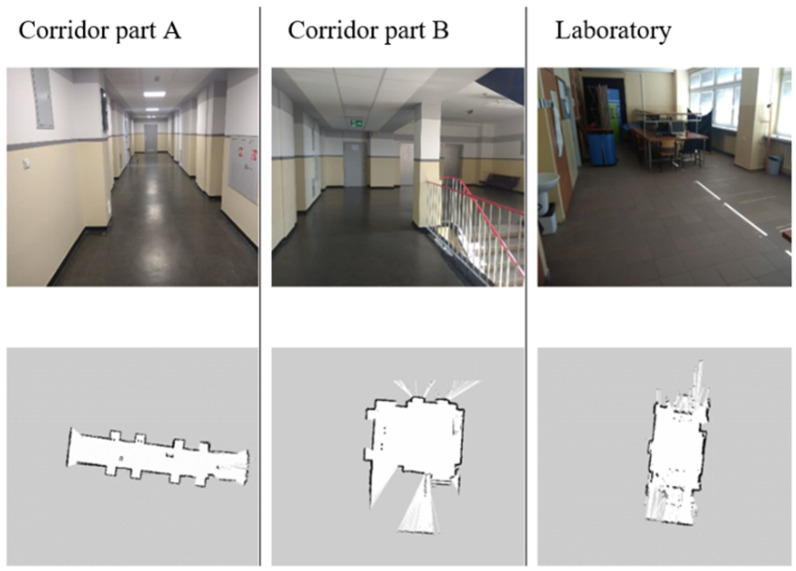
The test scenarios with the Lidar point map.

The tests were conducted in three measurement environments (Figure 12). Two of them were arranged in the corridor (part A—long narrow corridor, part B—section in front of elevators, space disturbed by the presence of a staircase). The third was located in the laboratory room (part C—space with many small objects, e.g., chairs, tables, laboratory equipment).

**Figure 13:**
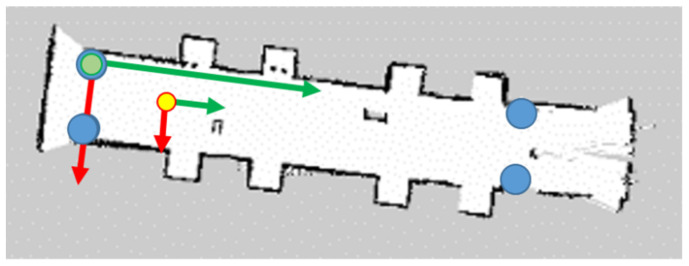
The distribution of the UWB system with a start point (yellow) and anchors (blue).

Figure 13 shows the distribution of UWB system tags during tests (blue circles), the external coordinate system associated with one of the tags (green circle), and the starting position of the platform (yellow circle). The initial position of the platform with respect to the local coordinate system was previously determined by the manual measure and was unchanged during tests.

UWB nodes configured as an anchor are used as a reference and are required for the UWB system to operate. Their location has been marked in relevant places on room maps (blue circles in Figure 13). As mentioned, data from tests were saved using the rosbag tool. This file contains logged data with timestamps, so it is possible to restore data traffic between nodes after tests for further processing. This approach greatly facilitates development results or creating algorithms through the opportunity to work only on saved data. The Rosbag tool additionally allows to divide the saved data file into smaller parts due to the length of the test or file size. The enrolment took place on the ODROID microcomputer.

## 6. Research Results and Discussion

**Figure 14:**
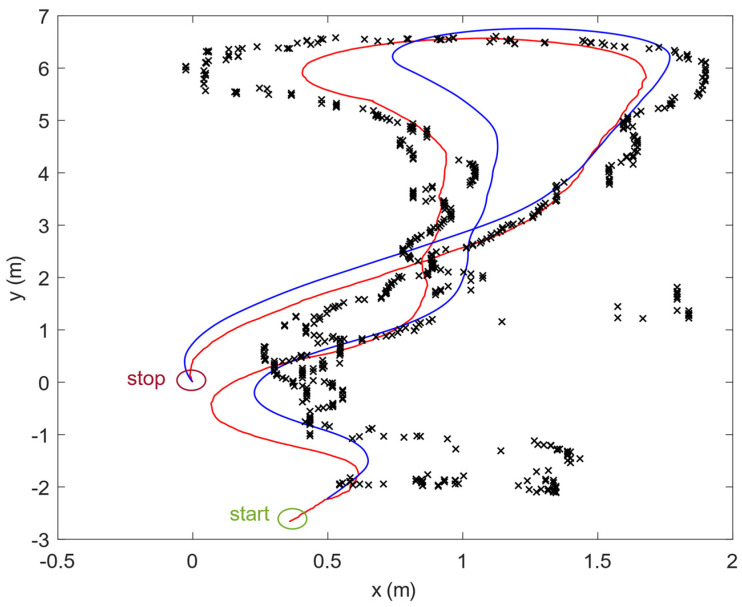
The trajectory determined by three methods in the environment “corridor part A.” Black points—UWB, red line—SLAM, blue line—dead reckoning.

Many tests have been performed indoors: on the corridor part A and B and the laboratory. The developed platform acquires the following data together with the timestamps for synchronization. Human has controlled the mobile platform, and the typical path/trajectory (2D view) is shown in Figure 14. The starting point is the bottom point of the line joining (the green square), and the stop point is above the start point (the red square). UWB raw data are shown as ‘x’ and the floorplan while the red line is SLAM (data from RPLidar). The reference path and the blue curve is obtained from encoders and myAHRS+ via dead reckoning.

The whole movement (corridor part A environment) took 31 s and allowed to collect about 1700 samples used for the dead reckoning (56 Hz data collection frequency), 590 sampled used in UWB positioning (19 Hz) and only 390 samples collected via SLAM (13 Hz).

**Figure 15:**
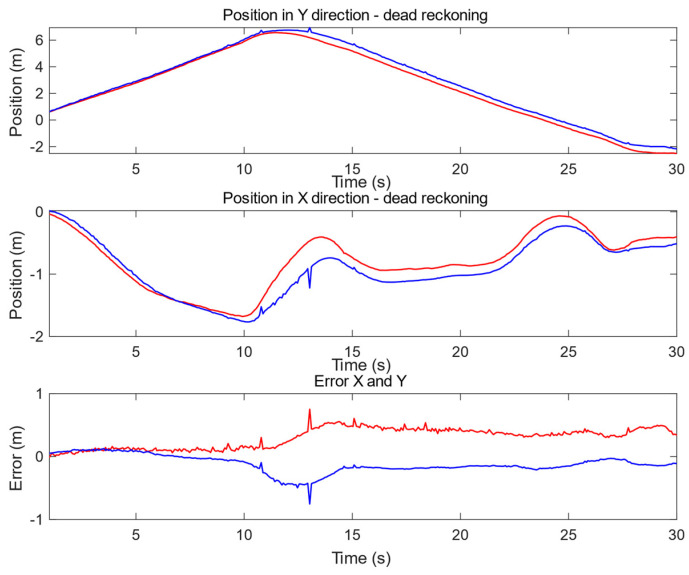
Plot present positioning results and error from dead reckoning in comparison to SLAM in the environment “corridor part A.” DR (blue) displacement with respect to SLAM (red): (**top**) vertical y position, (**middle**) horizontal X position, (**bottom**) DR error—displacement vector in horizontal (blue) and vertical (red) axes.

According to the exemplary trajectory, the error in X (horizontal direction) and Y (vertical direction) have been evaluated. The results are presented in Figure 15 (for corridor A) where three plots can be distinguished: (top) displacement based on encoder and myAHRS+ (in blue) with respect to the Y axis according to trajectory determined by SLAM (in red) from RPLidar (reference system); (middle) displacement based on encoder and myAHRS+ with respect to the X axis (colors as previously); the last plot (bottom) shows the dead reckoning displacement error for the Y axis (in red) and X axis (in blue).

**Figure 16:**
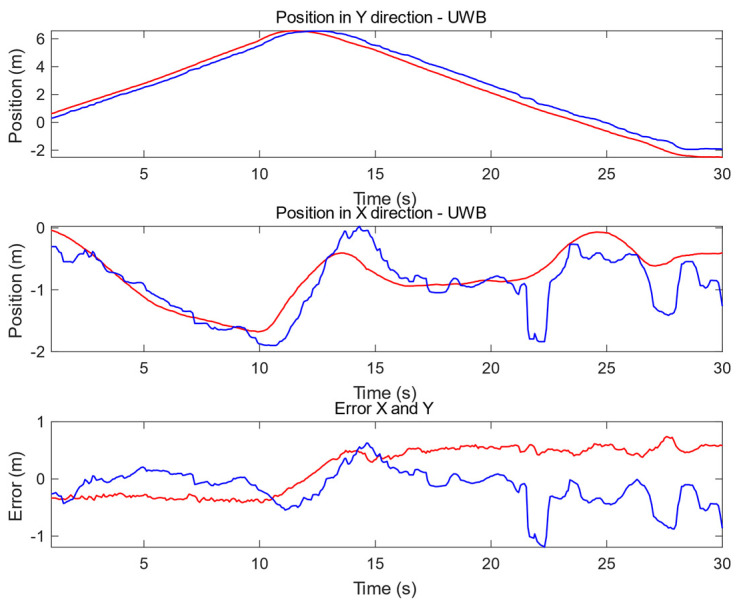
Plot present positioning results and error from UWB in comparison to SLAM in the environment “corridor part A.” UWB (blue) displacement with respect to SLAM (red): (**top**) vertical y position, (**middle**) horizontal X position, (**bottom**) error—UWB displacement vector in horizontal (blue) and vertical (red) axes.

Figure 16 presents another example for the path in corridor A for a comparison with DR (Figure 15), and presents three plots: (top) displacement based on UWB (in blue) with respect to the Y axis according to trajectory determined by SLAM (in red) from RPLidar (reference system); (middle) displacement based on UWB with respect to the X axis (colors as previously); (bottom) the last graph shows the UWB displacement error for Y axis (in red) and X axis (in blue).

The RMSE (root mean square error) and max deviation of the positioning using the UWB system and dead reckoning (without systematic correction) are presented in Table 2 separately for each environment and each axis.

When taking measurements in corridor part A, the UWB positioning system has problems due to the lack of coverage between the marker and reference point. Large metal objects such as the elevator doors near system operation can also affect the quality of the designated item. The RMSE of UWB positioning was about 1 m without using corrections. When the object is moving, it is better to rely on another positioning method and to use UWB as a complementary system.

The essential thing was the location of reference points and a tag. Antennas are directional, which hinders proper placement system components. When testing in the corridor near the elevators, it can be noticed that the positioning errors were higher when the mobile platform was closer to one of the reference points, but the other two were far away. The best results are obtained when the vehicle is stationary and can collect more data.

The data correction algorithm is described in Section 5, where data from one positioning system is corrected regularly using data from the more accurate system (but characterized by lower data rate). The error of the displacement using the UWB system and dead reckoning with systematic correction are presented in Table 3 separately for each environment.

The reference positions for correction are obtained from RPLidar. The displacement error (RMSE) as the time between corrections decreases. The average improvement (in reference to decreases no correction) is 23% when the corrections are made every 3 s and about 63% when the corrections are made every 1 s, no matter which positioning method was used (see Table 4).

Another important factor, the environment has a significant impact on the accuracy of measurements. The significant improvement in terms of periodic correction from RPLidar was noticed for the corridor part A and B, while the lowest in the laboratory class. This can be an effect of furniture which is made mainly of metal (chairs and laboratory tables). Such elements are not present in corridors. Taking into account corridors, the accuracy is much higher for part B than part A. The building structure, and a rather narrow part of corridor A (the width is appx. 3.5 m), seems not to be sufficient for UWB infrastructure placement (especially in case of deployment of anchors). Moreover, the corridor is long and may act as waveguide what reduces UWB system precision. The best results have been obtained for corridor part B for both dead reckoning and UWB systems. This is a result of UWB infrastructure placement as all anchors are not directly by the walls (like in corridor part A). These are the reasons why both systems (dead reckoning and UWB) require correction depending on the environment in which the measurements are carried out. On the other hand, in cases were environment conducive to the UWB system working conditions, both systems without correction give the highest possible accuracy. Without doubt, the RPLidar has the best conditions for simultaneous localization and mapping and gives the most accurate position. The results of the tests of the UWB system have been registered and are available in the form of a movie [31] (https://www.youtube.com/watch?v=-JsLYsDf_n0).

## 7. Conclusions

The research conducted shows the efficiency of the UWB positioning system in which accuracy can be increased by using RPLidar data for periodic correction. Both evaluated positioning systems, i.e., dead reckoning and UWB, have their advantages and disadvantages. The main advantage of using the only odometry is that no additional infrastructure is required for localization of the ego-vehicle. However, to detect other vehicles, a sensor like a lidar is necessary. The RPLidar seems to combine advantages: no additional infrastructure and precise perception of the environment, but there a lack of simultaneous communication and huge amounts of data to be processed for SLAM. The information from the RPLidar comes with an unspecified time interval that leads to surrounding gaps. Furthermore, SLAM needs some sort of landmarks for localization and does not work in sparse environments. The UWB positioning system is not as precise as RPLidar and the data are not so frequent as from odometry, it also requires infrastructure, but it allows for the use of offline maps and it also consists of an additional communication channel. For safety systems, like autonomous driving, the importance of redundancy is high. Thus, it is important to detect critical situations and determine the position using all three systems. In the case of relatively small differences in the determined position between SLAM and the dead reckoning or the UWB system, only SLAM can be used, while in other cases, a positioning system should rely on the dead reckoning, adjusted from time to time by measurements from UWB system for global localization.

Comparing UWB systems and dead reckoning, it can be stated that dead reckoning is more resistant to measurement noise, and its positioning better reflects the trajectories of the route. On the other hand, the error will increase over time as opposed to positioning using the UWB system. SLAM was used as a reference because it is the best way to determine the position of the platform, but only in places where there are enough characteristic points of the environment, i.e., indoors, but not necessarily outside.

Another big advantage in favor of using the UWB system is its greater resistance to work in an environment where the positioned object is obscured (if there are no conditions of direct LOS—a line of sight). In addition, since the UWB system works at a higher frequency than SLAM, it is possible to use more frequent position information for more detailed filtration, e.g., complementary filtration or Kalman filter. Further, the last advantage of the UWB is its communication capability that can be utilized in the new infrastructure across smart cities or to improve the quality of ADAS functionality. The entire research indicates also that indoor localization depends on the environment and it requires the positioning algorithm to include a method for recognizing the environmental conditions in which the measurements take place.

## Figures and Tables

**Table 1:** Advantages, disadvantages and parameters related to devices used in the RC platform and related systems.

	UWB	IMU	RPLidar
Positioning algorithm	Trilateration	Dead reckoning	SLAM
sampling rate	19 Hz	56 Hz	13 Hz
Device limitations e.g., coverage and other parameters	Up to 40 m × 40 m, Max 5 tracked devices appx. 50 readings per sec for 2 device setup	Coverage not applicable, Triple axis 16-bit gyroscope Triple axis 16-bit accelerometer accuracy ±0.2°. Triple axis 13-bit magnetometer	Coverage not applicable (depends on surrounding environment), Measuring range: 0.15–12 m Accuracy: <1% of distance Distance resolution: 1 mm Angular resolution: 0.9° Time of single measurement: 0.25 ms
Advantages	• High accuracy • Not dependent on the surface or experiment setup. Coverage 100%	• High data rate • Not dependent on environment setup • Can be used in open areas Coverage 100%	• All positions are determined independently • Moderately high data acquisition frequency Possible V2V data transmission
Disadvantages	• The surroundings should not be changed rapidly There must be enough characteristic points around to achieve localization	• Accumulate error over time • Tire slip errors • Reference point is required	• Requires setup which is not possible everywhere • Need infrastructure Coverage depending on infrastructure

**Table 2:** The positioning errors using different methods.

Environment	Error [m]	Dead Reckoning		UWB	
		X	Y	X	Y
corridor part A	Max	0.75	0.75	1.19	0.74
	RMSE	0.18	0.33	0.38	0.44
corridor part B	Max	0.13	0.43	0.88	1.35
	RMSE	0.05	0.24	0.43	0.65
laboratory	Max	1.41	0.87	1.18	0.66
	RMSE	0.15	0.24	0.44	0.33

**Table 3:** The displacement errors using different methods without and with regular correction.

Environment	Correction	Dead Reckoning		UWB	
		Max [m]	RMSE [m]	Max [m]	RMSE [m]
Corridor part A	No	0.84	0.34	1.21	0.41
	Every 7 s	0.83	0.27	0.95	0.28
	Every 3 s	0.74	0.20	0.78	0.25
	Every 1 s	0.53	0.08	0.57	0.15
Corridor part B	No	0.30	0.13	0.85	0.34
	Every 7 s	0.33	0.11	0.67	0.31
	Every 3 s	0.47	0.11	0.67	0.27
	Every 1 s	0.28	0.03	0.47	0.13
Laboratory	No	0.85	0.16	0.99	0.37
	Every 3 s	0.93	0.14	1.36	0.34
	Every 1 s	1.08	0.10	0.78	0.15

**Table 4:** The improvement using systematic correction.

Environment	Correction	Dead Reckoning	UWB
corridor part A	Every 7 s	21%	32%
	Every 3 s	41%	39%
	Every 1 s	76%	63%
corridor part B	Every 7 s	15%	9%
	Every 3 s	15%	21%
	Every 1 s	77%	62%
laboratory	Every 3 s	13%	8%
	Every 1 s	38%	59%
Average	Every 7 s *	18%	21%
	Every 3 s	23%	23%
	Every 1 s	64%	62%

* only for corridor part A and B.
